# Harnessing the Natural Toxic Metabolites in COVID-19

**DOI:** 10.1155/2022/3954944

**Published:** 2022-03-07

**Authors:** Ali Bahrami, Mohammad Taheri, Mohammad Reza Arabestani, Meysam Soleimani, Mojdeh Mohammadi, Fatemeh Golabchi, Maryam Banitorfi, Seyed Mostafa Hosseini, Sodabe Khodabandehlou, Fatemeh Nouri

**Affiliations:** ^1^Student Research Committee, Hamadan University of Medical Sciences, Hamadan, Iran; ^2^Department of Pharmaceutical Biotechnology, School of Pharmacy, Hamadan University of Medical Scienceaffs, Hamadan, Iran; ^3^Department of Microbiology, Faculty of Medicine, Hamadan University of Medical Sciences, Hamadan, Iran; ^4^Department of Pharmacology and Toxicology, School of Pharmacy, Hamadan University of Medical Sciences, Hamadan, Iran

## Abstract

SARS-CoV-2 is a novel coronavirus and the cause of the recent pandemic; it is an enveloped *β*-coronavirus. SARS-CoV-2 appear in the Wuhan City of China for the first time and outspread worldwide quickly. Due to its person-to-person fast transmission, COVID-19 is becoming a global problem. SARS-CoV-2 enter into cells by using ACE2 receptors that are numerous in the lungs and finally can cause acute respiratory distress syndrome (ARDS). Dry cough, sore throat, fever, body pain, headache, GIT discomfort, diarrhoea, and fatigue are some of the COVID-19 symptoms. There is no definite and certain treatment for disease caused by SARS-CoV-2 till now. Some pharmacological effects of toxins, toxoids, and venoms have been proven, and their effects on some diseases have been evaluated. This study aimed to investigate the role of toxins, toxoids, and venom in the pathophysiology of COVID-19 disease.

## 1. Background

Acute respiratory syndrome coronavirus 2 (SARS-CoV-2) is characterized by severe cytokine syndrome following inflammation. The pathogenesis of SARS-CoV-2 is such that in the first stage, viral binding to epithelial cells occurs with a minimal innate immune response. In the second stage, we see increased viral replication along with an active immune response and the spread of the virus to the lower respiratory tract, which may also affect the digestive and cardiovascular systems. In the third stage, events such as hypoxia, the penetration of the virus into the entire respiratory tract, and finally acute respiratory distress syndrome (ARDS) occur, which the fatal rate is potentially high [1].

SARS-CoV-2 affects the lungs the most, but it can also attack the brain and cause unpredictable nervous defects by crossing the blood-brain barrier [2]. SARS-CoV-2 is transmitted person-to-person through viral airborne droplets. This infection has several clinical symptoms such as dry cough, sore throat, fever, body pain, headache, abdominal discomfort, diarrhoea, and fatigue. In the acute phase, it can cause pneumonia, acute respiratory failure, encephalopathy, multiorgan dysfunction, and death. Because angiotensin-converting enzyme 2 (ACE2) is one of the major receptors identified for SARS-CoV-2 and is predominantly expressed in the lungs, the lungs are involved in coronavirus infection. This receptor can also be found in the gastrointestinal tract, the cardiovascular system, the brain, and other organs [3–5].

Various data and treatments for SARS-CoV-2 infection are being reviewed. We previously examined the impact of serum electrolytes, trace elements, and heavy metals in COVID-19 [6, 7]. Toxins are small molecules, peptides, or proteins secreted by prokaryotic and eukaryotic cells and provide defence ability for them [8]. Phospholipases, proteases, and ion channel modulators are their primary targets. Many toxins are multifunctional and have several biological targets which may have no relation with their toxic role [9]. Some toxin-derived peptides are now being used to treat type 2 diabetes, hypertension, neuropathic pain, and other medical disorders [10–13]. Some data confirm the effect of bee venom (BV) on preventing COVID-19 and improving it [1]. Some other data ignore the BV effect on preventing COVID-19 and hypothesize that less SARS-CoV-2 infection in beekeepers is due to their less exposure to other people [3]. A low dose of botulinum neurotoxin (BoNT) can reduce the symptoms of COVID-19, and so, it could be used in treatment lines [14]. It has demonstrated that the rate of DTP vaccination has an inverse correlation with COVID-19 prevalence [15]. Cobrotoxin has an anti-inflammatory effect and also can restore the CD4/CD8 ratio and perform immunoprotective activity against SARS-CoV-2 [16]. Tetrodotoxin is an inhibitor of M^Pro^ of SARS-CoV-2 and so can affect the virus [17]. This study aimed to investigate the role of toxins, toxoids, and venom in the pathophysiology of COVID-19 disease.

## 2. Method

**Figure 1:**
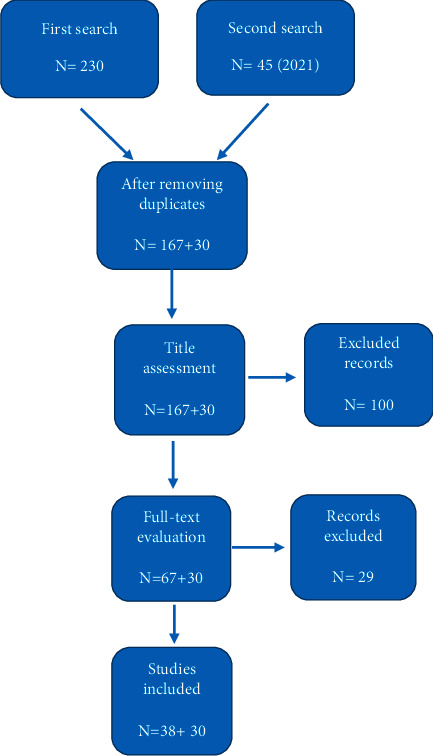
Flow chart of the search strategy.

The bibliographic search was performed on PubMed, Scopus, and Web of Science databases in Sep 2021. Search keywords including “toxin” OR “aflatoxin” OR “botulinum toxin A” OR “dioxin” OR “diphtheria toxin” OR “exotoxin” OR “pertussis toxin” OR “tetanus toxin” OR “tetrodotoxin” OR “trichothecenes” OR “Shiga toxin” OR “cholera toxin B” OR “bufotoxin” OR “ochratoxin A” OR “Anthrax toxin” AND “coronavirus 2019” OR “COVID-19” OR “SARS-CoV-2” in all fields. Any languages or date restrictions were not applied. Identified studies were screened by title, abstract, and full text. The reference list of identified studies was also evaluated to increment the sensitivity and choice of most literature which we could not identify in the database. Greatest sensitivity search was initiated, 167 articles on external databases were found and collected by a researcher using the Endnote software. Then, unifying the articles from all the cited databases and bringing out duplicate articles, the two researchers separately investigated all the articles and excluded the articles that includes irrelevance of title to toxins and COVID-19, absence of keywords in the title or abstract of studies, and the inclusion index criteria. Afterwards, several articles after reviewing titles and abstracts were excluded. The existing publications were carefully assessed, the relevant study was chosen, and the data for the studies were appraised based on the title, examined technique, sample size, and so on. Finally, 38 articles were analyzed after acquiring relevant papers and determining the limitations of the search strategy. During the examination of publications in 2021, we discovered 30 new articles that were included in the study (search strategy was shown in Figure 1).

## 3. Results

### 3.1. Bee Venom

Apitherapy means the use of bee stings to treat certain diseases. However, the results regarding the effectiveness of this method are controversial [3]. Bee venom (BV) has antiviral properties because of the presence of melittin and phospholipase A2 (PLA2). Both agents work against enveloped and nonenveloped viruses, and they work against H1N1 and HIV by using antagonist activity against IL-6, IL-8, interferon, and TNF-*α*. BV vaccination is also a way to protect against cytomegalovirus. In modern medicine, bee venom is used to treat Parkinson's disease, multiple sclerosis, and arthritis [1, 18–20].

According to a study in Hubei Province in China, none of the 5115 local beekeepers had covid-19 symptoms. 5 apitherapists and 121 patients who received apitherapy were also interviewed. Two of the apitherapists, although exposed to suspected or confirmed covid-19 victims without protection, showed no signs of COVID-19. Also, none of the 121 patients was infected with COVID-19, even though 3 of them were exposed to relatives infected with SARS-CoV-2. They suggested that BV therapy, due to the presence of melittin and phospholipase A2, which have a strong anti-inflammatory function, could help support recovery because even if the patient recovers from the initial SARS-CoV-2 infection, and it may have long-term effects which are known as long-covid because PLA2 binds to the membrane *in vivo*, causes antibodies to bind with the cell membrane, and stimulates CD8 T cells [1]. Bee venom increases the differentiation of FOXP3-expressing cells in CD4 T cells and mature CD4 thymocytes [21, 22]. Yang et al. believe that the effect of BV on the immune system and increasing the differentiation of human regulatory T cells plays an important role in controlling COVID-19 [18]. Also, Block et al. believe that the anti-inflammatory and antimicrobial properties of BV derivatives may help prevent long-term fibrotic destruction of the lungs. It has demonstrated that IL-10 increase in beekeepers due to their persistent exposure to BV. IL-10 is an anti-inflammatory cytokine that reduces inflammatory cytokines IL-1 and TNF-alpha and so, BV can decrease the cytokine storm [3, 23].

A study conducted in Germany did not confirm the results of apitherapy in SARS-CoV-2. The study found that beekeepers were not immune to SARS-CoV-2 infections. The hypothesis that beekeepers will not be infected with SARS because of immunity due to bee stings is not supported by the data of this study. Some factors like how long they had been a beekeeper, the total number of bee stings received, the number of bee stings received in the year 2020, and potential allergic reactions to bee stings do not have any effect on the severity of COVID-19. However, beekeepers' reactions to bee stings are one of the elements that influence two COVID-19 symptoms, including tiredness and sore throat soreness. These symptoms are more pronounced in beekeepers who are more sensitive to bee stings. The fact that beekeepers are less affected by SARS-CoV-2 infection may be due to their personality traits, which tend to spend more time alone and therefore less exposed to contact with other humans and COVID-19. BV and the melittin it contains can regulate Th1 and other immune cells and are used to lower viral load and reduce the severity of interstitial pneumonia in PRRSV-infected pigs. These effects may also be crucial in the case of SARS-CoV-2 pneumonia, but these effects were only achieved when BV was administered through the nasal or rectal route. The various effects of BV on the immune system and its resistance to SARS-CoV-2 should be considered as an indicator of the level of the immune response, rather than as a definite therapy strategy [3].

### 3.2. Botulinum Neurotoxin (BoNT)

The use of therapeutic botulinum neurotoxin (BoNT) against SARS-CoV-2 is also being considered. Botulinum toxins are powerful neurotoxins that can cause muscle paralysis and acute respiratory arrest in humans, but a mild dose of the pure form of Botox therapy is known to reduce the common clinical symptoms of COVID-19 including chronic cough, dyspnoea, pneumonia, acute respiratory failure, abnormal circulation, cardiac defects, and various neurological deficits. A low dose of purified BoNT is also used in many diseases such as strabismus, blepharospasm, chronic migraine, and overactive bladder. Therapeutic BoNT can be used as a method of preventing SARS-CoV-2 infection in high-risk populations. To reduce its side effects, antioxidants can also be used along with treatment. It reported that the side effects of therapeutic BoNT are temporary and reversible, and the action of this toxin in very mild doses is relatively safe [24]. There are some reports that therapeutic BoNT in very mild doses can suppress many human diseases by 10 to 20 units [25–28]. It also can migrate from the site of intramuscular injection to the brain and other organs. The beneficial effects of a single therapeutic dose are probably long-lasting. Designing a multifaceted drug to fight SARS-CoV-2 infection can be effective, and therapeutic BoNT can be a good option [14]. In a study in France, 193 patients who used BoNT/A in different doses for different purposes such as migraines and facial palsy were evaluated, and a critical contrast between the number of infected people within the common populace and the number of patients injected with BoNT/A who appeared signs of COVID-19 has seen [29].

### 3.3. Tetanus and Diphtheria

There is evidence suggesting that a tetanus toxoid vaccine could reduce the severity of COVID-19 symptoms. A connection between tetanus toxin and the COVID-19 spike glycoprotein as well as a similar connection between the tetanus toxin protein sequence and other coronaviruses was observed [30]. Also, neurological symptoms and temporomandibular joint (TMJ) pain, which are common symptoms of tetanus disease, have been reported in several COVID-19 patients. Also, in comparison between the United States and other countries where tetanus vaccination rates were lower, an inverse correlation was observed between tetanus vaccination rates and mortality rates [31, 32]. We know the DTP vaccine contains toxoids of tetanus, diphtheria, and pertussis, and it is noteworthy that there is a correlation between the rate of DTP vaccination in children worldwide and the rate of asymptomatic COVID-19. A similar association is observed in pregnant women, who are advised to receive two flu and TDaP vaccines in the third month of pregnancy [30]. As a result, it may be inferred that tetanus and diphtheria vaccines may have a protective effect against COVID-19 in both children as well as adults who are up-to-date on their tetanus vaccination. Two other studies also noted a reduction in the severity of COVID-19 in children and its possible association with DTP vaccination and suggested that the DTP vaccine could stimulate the immune system. In one case, it was concluded that the DTP vaccine is the only vaccine with a high potential for cross-reactivity with COVID-19 spike protein [15, 33]. CRM197, a modified diphtheria toxin found in the Hib vaccine, and rubella vaccine are highly similar to SARS-CoV-2 proteins, suggesting that they may have some anti-SARS-CoV-2 activity [34].

### 3.4. Cobrotoxin

Cobrotoxin, a short-chain *α*-neurotoxin from Naja naja atra venom (NNAV), may be effective in treating COVID-19 patients and inhibiting SARS-CoV-2 infection. The inflammatory cytokine storm causes aggravation of lung disease in COVID-19 patients, in addition to other deadly consequences. As a result, in addition to attempting to restrict virus replication, anti-inflammatory medication is an important method of combating COVID-19 disease. Cobrotoxin and alpha-neurotoxins have anti-inflammatory activity and prevent the binding of the nuclear factor-*κ*B to DNA, which is a transcription factor that regulates the expression of genes involved in the inflammatory response, thereby reducing the transcription of inflammatory genes. Cobrotoxin inhibits CD8 T cell proliferation more than that of CD4 T cells, and since COVID-19 cellular immune responses are induced by overexpression and hyperactivation of cytotoxic T lymphocytes [35], cobrotoxin can restore the CD4/CD8 ratio and perform an immunoprotective activity [16].

### 3.5. Tetrodotoxin

Tetrodotoxin is a sodium channel blocker and acts on the nervous system message delivery [36]. The toxicity of tetrodotoxin is 1200 times more than cyanide. One of the important drug targets in coronaviruses is the main protease (M^Pro^) due to its important role in controlling replicate complex activity and its vital role in viral replication and transcription [37, 38]. Tetrodotoxin is a potential M^Pro^ inhibitor of SARS-CoV-2 which may be a potential compound against SARS-CoV-2 according to the results of the ligand-based approaches [17].

### 3.6. Snake Venoms

Nature is known to offer numerous biotherapeutics from animal venoms, green growth, and plant that have been generally utilized in conventional medication. Among these bioresources, snake poison shows numerous bioactivities such as antiviral, antiplatelet, antithrombotic, anti-inflammatory, antimicrobial, and antitumoral. Snake venom contains a mixture of amino acids, proteins, peptides, nucleotides, lipids, carbohydrates, and metal elements along with proteins [39, 40]. Several studies have reported that snake venom ingredients have antiviral activity against measles, Sendai, dengue, yellow fever, and human immunodeficiency virus [41–43]. Snake venoms are a complex combination of proteins and peptides. Venom serine proteases (SVSPs), snake venom metalloproteinases (SVMPs), secreted phospholipases A2 (SV-PLA2s), C-type lectins, and disintegrins are the major groups of snake venom components, while the minor group includes nucleotidases (Ntases), phosphodiesterases (PDEs), cysteine-rich secretory proteins, L-amino acid oxidases, Kunitz peptides, three-finger peptides (3FTX), and natriuretic peptides [44–48]. Bradykinin-potentiating peptide 10C (BPP-10C) isolated from *Bothrops jararaca* can decrease angiotensin II by inhibiting the ACE and increase bradykinin 2-receptor [49–51] that both have a role in the pathogenesis of SARS-CoV-2 [52–55], and it can be considered as an anti-SARS-CoV-2 agent [56]. Kunitz-type peptides ((also called bovine pancreatic trypsin inhibitors) (BPTIs)) are 50 to 60 amino acid components found in the snake venoms that inhibit the catalytic site of serine proteases [57]; so, they are potential antiviral agents because transmembrane protease serine‐2 (TMPRSS2) activity is required for SARS-CoV-2 entry [58, 59]. Clinical information has appeared that numerous patients with serious COVID-19 display coagulation anomalies such as microvascular thrombosis and venous or blood vessel thrombosis [60–63]. Snake venoms are wealthy sources of bioactive particles interfering with the blood coagulation cascade and platelet aggregation [64, 65]. Phospholipase A2s (PLA2s) are one of the main components of snake venoms [66]. HDP-1 and HDP-2 are PLA2s that are isolated from *V. nikolskii* and could inhibit SARS-CoV-2 binding to ACE2 [67]. So, they can be effective in improving COVID-19 [68].

## 4. Discussion

Coronavirus pandemic induced by SARS-CoV-2 has been the cause of high burden worldwide. On 4 February 2022, the WHO announced 383,510,000 confirmed coronavirus illness 2019 cases and 5,700,000 deaths due to COVID-19. Although wide research has been carried out on the finding of effective therapeutics, so far there are no approved treatments against COVID-19. In the present study, harnessing natural toxic metabolites have been prioritized to make a review focusing on the efficacy of these metabolites as therapeutic agents [69]. Chloroquine/hydroxyl chloroquine combined with azithromycin had been used to improve COVID-19, but the results were controversial [70]. Remdesivir was the only approved antiviral drug for COVID-19 that was administrated for severe patients [71]. Some evidence indicates that toxins, toxoids, and venoms might be effective against SARS-CoV-2 through their anti-inflammatory, M^Pro^ inhibitory, or antiviral effect. Due to the presence of melittin and phospholipase A2 (PLA2) in BV, it has antiviral effects on some enveloped and nonenveloped viruses. We know that after curing COVID-19, also some long-term effects may remain. BV has also a positive effect on recovery from long-COVID-19 [1]. Another idea is that fewer infected beekeepers are due to their less contact with other people and not for the effect of BV [3]. A low dose of pure botulinum toxin can reduce some symptoms of COVID-19-like acute respiratory failure and dyspnoea [14]. Tetanus toxin is similar to SARS-CoV-2 spike proteins and has been demonstrated that there is a correlation between DTP vaccination and the severity of COVID-19. Also, the rate of COVID-19 infection in pregnant women who received the TDaP vaccine decreases [30]. CRM197 that is a modified diphtheria toxin is similar to SARS-CoV-2 proteins and is present in some vaccines such as Hib and rubella. So, these vaccines may also have a protective effect on COVID-19 [34]. Cobrotoxin regulates the expression of genes involved in the inflammatory response and can prevent cytokines storm that causes more lung damage. Also, CD4/CD8 ratio that is imbalanced in COVID-19 can restore by cobrotoxin [16, 35]. Tetrodotoxin inhibits main protease (M^Pro^) and affects the replication of the virus [17]. Toxins, toxoids, and venoms may have effects on SARS-CoV-2 disease and prevent disease or reduce its symptoms. In addition, to achieve definitive results and efficacy of natural toxic metabolites comprehensive studies are needed (the data summary is presented in Table 1).

## 5. Conclusion

In this study, we investigated the role of toxins, toxoids, and venom on the pathophysiology of COVID-19 as an inflammatory disease. We discussed BV, botulinum toxin, tetanus toxin or toxoid, diphtheria toxin or modified toxin, cobrotoxin, and tetrodotoxin, which can be impressive in the pathophysiology of COVID-19. More surveys and clinical assessments are needed for better knowledge about the effect of toxins on COVID-19.

## Figures and Tables

**Table 1:** Data summary.

Bee venom	IL-1 and TNF-*α* decrease because of an increase in cytokine IL-10 due to bee venom, so, cytokines storm could be weakened. Also, bee venom contains melittin and phospholipase A2 that have an antagonist effect on IL-6, IL-8, interferon, and TNF-*α*. [1, 3, 18–20, 23]. Less infection in bee keepers is due to their less exposure to other people not because of bee venom [3].
Botulinum neurotoxin (BoNT)	A mild dose of the pure BoNT can reduce the common clinical symptoms of COVID-19 including chronic cough, dyspnoea, pneumonia, acute respiratory failure, abnormal circulation, cardiac defects, and various neurological deficits [14].
Tetanus and diphtheria	Tetanus toxin and diphtheria toxin both are similar to SARS-CoV-2 proteins, and so, the administration of the DTP vaccine may have a protective effect against SARS-CoV-2 [30, 34].
Cobrotoxin	Cobrotoxin prevents the expression of inflammatory genes and also balances the ratio of CD4/CD8 cells and performs immunoprotective activity [16].
Tetrodotoxin	Tetrodotoxin is a potential M^Pro^ inhibitor of SARS-CoV-2. So, it may affect SARS-CoV-2 [17].

## Data Availability

The data that appeared in this study are already publicly available in the literature.
